# Formation of giant iron oxide-copper-gold deposits by superimposed episodic hydrothermal pulses

**DOI:** 10.1038/s41598-023-37713-w

**Published:** 2023-07-25

**Authors:** Irene del Real, Martin Reich, Adam C. Simon, Artur Deditius, Fernando Barra, María A. Rodríguez-Mustafa, John F. H. Thompson, Malcolm P. Roberts

**Affiliations:** 1 Institute of Earth Sciences, Austral University, Valdivia, Chile; 2 Department of Geology, FCFM, Universidad de Chile, Plaza Ercilla 803, Santiago, Chile; 3 Department of Earth and Environmental Sciences, University of Michigan, Ann Arbor, MI USA; 4 Chemistry and Physics, Murdoch University, Perth, WA 6150 Australia; 5 PetraScience Consultants, 3995 West 24th Avenue, Vancouver, BC V6S 1M1 Canada; 6 Centre for Microscopy, Characterization and Analysis (CMCA), University of Western Australia, Perth, WA 6009 Australia

**Keywords:** Geochemistry, Solid Earth sciences, Economic geology

## Abstract

Iron oxide-copper-gold (IOCG) deposits are a globally important source of copper, gold and critical commodities. Despite their relevance, IOCG deposits remain an ill-defined clan, with a range of characteristics that has complicated development of the general genetic model. Here we focus on the Candelaria IOCG deposit in Chile and reveal that by using micro-textural and compositional variations in actinolite, a common alteration mineral found in many IOCG deposits, we can constrain the evolution of these systems. We demonstrate that Candelaria formed by the superposition of at least two pulses of mineralization with a late Cu-rich event overprinting and superimposed over an early, and probably higher temperature, iron oxide-apatite (IOA) mineralization event. These distinct pulses were likely caused by episodic injections of magmatic-hydrothermal fluids from crystallizing magmas at depth. Our data provide empirical evidence of grain-to-deposit scale compositional and potentially temperature changes in an IOCG system. The results support the use of actinolite chemistry as a novel approach to understand the formation of IOCG deposits and a potential tool for vectoring in exploration.

## Introduction

Iron oxide-copper-gold (IOCG) systems are among the world’s richest mineral deposits, making for highly profitable mining operations^1–3^. By-product strategic elements including Co, U and Rare Earth Elements (REE) add to their attractiveness. Previous studies on important IOCG deposits such as Olympic Dam (Australia), Candelaria (Chile), and Salobo and Sossego (Brazil) have shown that they are closely related to basement penetrating fault systems^1,2^, and have undergone an early magnetite-actinolite event followed by main-stage mineralization of copper and other elements^4–6^. Even though there is abundant evidence for magmatic signatures in IOCG deposits—Geochemical and isotopic^1,3,7^—Most of these deposits show no apparent genetic relationship with exposed coeval igneous intrusions^1–3,7^. Intrusions are inferred at depth but the magmatic events leading to mineralization are not well defined. IOCG deposits also lack substantial quartz veining which has hindered the use of fluid inclusion and related studies to determine the temperature and composition of the mineralizing fluid(s)^8–12^. Geochemical proxies, particularly the chemical composition and Fe-O-H-S isotopic signatures of selected silicates, magnetite and pyrite, have provided some insights into the nature of the hydrothermal fluids^13–22^, but the thermal evolution of IOCG systems in space and time remains poorly constrained. In particular, the thermal and spatial evolution constraints of early Fe-rich and the main Cu(-Au) mineralization stage have not been addressed. Understanding this relationship is critical to development of a universally applicable genetic model.

One of the most common minerals found in both (early) Fe-rich and (later) Cu-rich mineralization stages in IOCG deposits is actinolite, (Ca_2_)(Mg_4.5–2.2_Fe_0.5–2.5_)(Si_8_O_22_)(OH)_2_. Actinolite has been well reported in the mineral paragenesis of Andean IOCG deposits, including Candelaria, Mina Justa and El Espino^6,23–25^. Therefore, the chemical composition of actinolite can potentially be distinct between early and later mineralization stages in IOCG deposits, and consequently be used as a proxy for characterizing their temporal evolution. The chemical composition of actinolite has been previously used for determining temperature conditions in iron oxide-apatite (IOA) deposits, where experimental data demonstrate that the thermal stability of amphibole depends on its Fe# (Fe#=*X*_Fe_/[*X*_Fe_+*X*_Mg_]; concentrations are in atomic %), which changes over a wide P-T range^26,27^.

In this contribution, we examine micro-textural and compositional variations of actinolite to determine the evolution of the Candelaria IOCG deposit. The Candelaria deposit is part of the Candelaria-Punta del Cobre district and is located in the Mesozoic Andean IOCG belt, one of the most fertile copper provinces in the world. The deposit has not been modified substantially by post-formation processes, and hence provides an ideal setting to perform a detailed examination of actinolite recovered along a ~1 km long drill core that cross cuts the entire mineral system. Our results were able to identify two chemically distinct groups of actinolites that represent an early Fe rich and a later Cu-rich mineralization pulse and provide empirical evidence indicating a key role for successive magmatic fluid injections in the formation of IOCG deposits and yields new insight to how IOA and IOCG deposits relate.

### Geology of the Candelaria-Punta del Cobre district

The Candelaria-Punta del Cobre district is located south of the city of Copiapó in northern Chile and comprises more than nine active IOCG mines, all interpreted to be part of the same hydrothermal system^6^ (Fig. SM1, Supplemental Material). IOCG deposits in this district are spatially and temporally associated with a north-northwest sinistral fault system and are interpreted to be coeval with a northwest transpressive deformation^6^. Copper mineralization is predominantly hosted in the Lower Cretaceous (~135–132 Ma) volcanic-sedimentary Punta del Cobre Formation that is overlain by sedimentary marine sequences from the Lower Cretaceous (132–130 Ma) Chañarcillo Group^6,28^. District-wide, early calcic-sodic alteration is observed towards the northwestern side of the district followed by widespread magnetite-actinolite alteration that formed between ~120–116 Ma^6^. This early magnetite-rich alteration extends beyond all deposits in the district (both in depth and laterally) and is observed as disseminated and pervasive magnetite-actinolite that can completely replace the volcanic host rocks, this style of alteration is common in IOA deposits (e.g. Dominga^29^, Marcona^30^).

**Figure 1:**
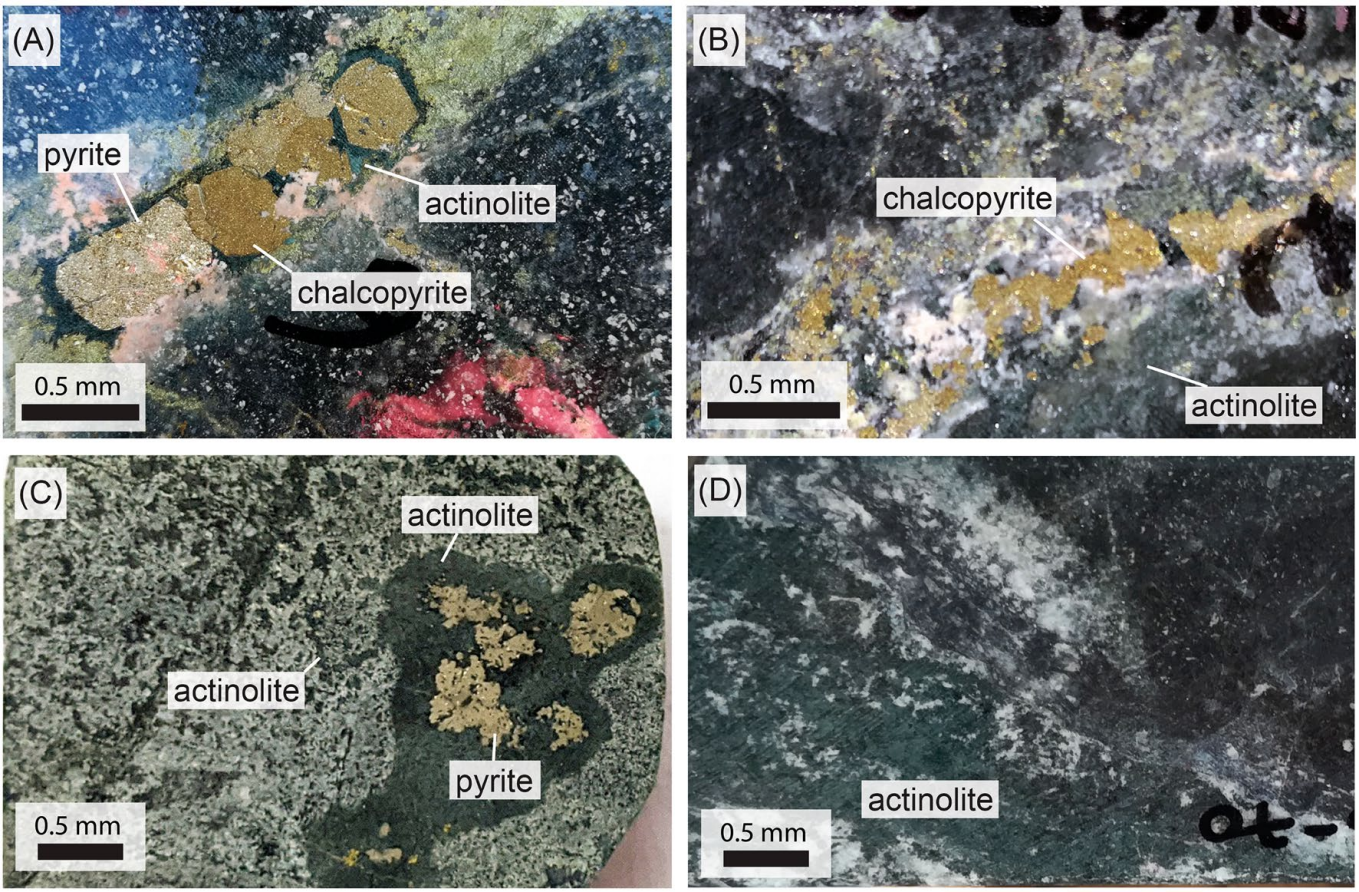
Photos representing pre and syn-mineralization actinolite alteration within the Candelaria deposit. Photo (**A**) corresponds to sample LD1687-13 (depth 203.6m) where actinolite is intimately associated with sulfide mineralization. Photo (**B**) corresponds to sample LD1687-35 (depth 585.12m) where actinolite is observed close to sulfide mineralization but not clearly coeval. Photo (**C**) corresponds to sample LD1687-63 (depth 1001.3m) where actinolite is observed finely disseminated in the host rock and in a cumulate surrounding pyrite. Photo (**D**) corresponds to sample LD1687-70 (depth 1109.5m) where actinolite is observed as a pervasive alteration together with magnetite and with no sulfides. Further details for each sample can be found in Table SM1 (supplemental material).

In the Candelaria deposit, mineralization and related alteration are hosted mainly in the lower member of the Punta del Cobre Formation to depths in excess of 800 m where the early magnetite-actinolite and calcic-sodic alteration stages are overprinted by a biotite–K-feldspar–chalcopyrite ± magnetite–actinolite alteration stage. This alteration and the associated main Cu mineralization event is dated at ca. 115 Ma^6,8^. The main ore body in the Candelaria deposit is up to 400m thick in the central part of the deposit and thins towards the margins^6,8^. Actinolite occurs in veins together with massive granular aggregates or disseminations of magnetite or sulfides in the volcanic host rocks. In all its forms, actinolite is a common alteration mineral in the Candelaria deposit^6^ that is typically associated or with Cu mineralization (Fig. 1A,B and C) or with the early magnetite event (Fig. 1C and D), indicating that it formed both during pre- and syn-mineralization stages. Both early and main stage events in the Candelaria deposit, and mineralization elsewhere in the district, are broadly coeval with the emplacement of the Copiapó Batholith located west of the main deposits^31^, but no field evidence has been documented to suggest that hydrothermal fluids for the early or main Cu mineralizing events were sourced directly from the batholith^6^.

## Methods

**Figure 2:**
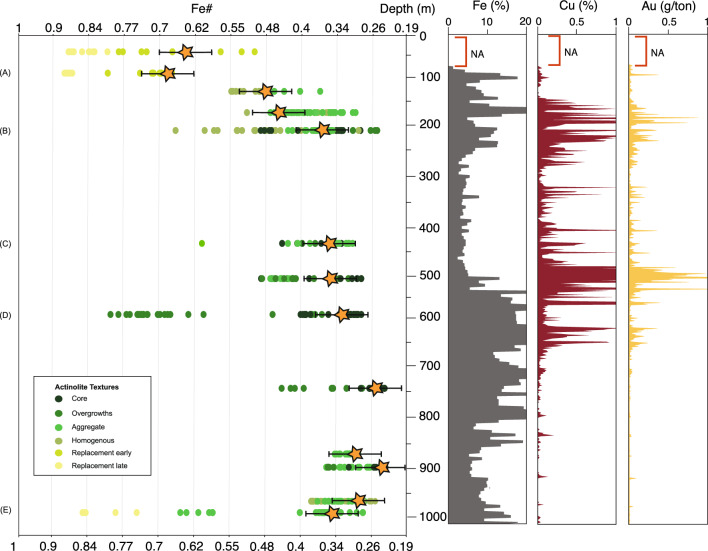
Calculated Fe# of actinolite plotted as a function of depth in drill core LD1687. Letters (**A**) to (**E**) indicate samples with calculated Fe# distribution maps (see Fig. 3). Letters correspond to the maps in Fig. 3. The vertical plots display variation of the Fe, Cu and Au contents with depth that were calculated from whole rock from half drill core samples 2m long (Lundin Mining, pers. comm.). Orange stars correspond to average Fe# values from early paragenetic textures (e.g. grain cores).

Samples for this study were collected from a 1000 m long drill core that traverses the main Cu orebodies and underlying magnetite-bearing altered rocks at Candelaria (Fig. 2). Actinolite was sampled systematically throughout the length of the drill core from both early and main mineralizing episodes providing a comprehensive and representative sample set (full description in Table SM1, Supplemental material). In areas with Cu mineralization, distinguishing petrographically among pre and syn-mineralization actinolite at the hand sample scale is challenging, as there is no obvious textural difference between the two stages. However, backscattered electron (BSE) images of the analyzed samples reveal significant micro-textural variations, including actinolite grains with marked core-to-rim chemical zoning, actinolite overgrowths on earlier actinolite, chemically homogenous actinolite, and small crystal aggregates (Fig. SM2, Supplemental Material). The different textures identified through BSE images reflect the relative timing for different actinolite grains (e.g. overgrowths or replacement) but there is no clear relationship between these texture and early vs syn-mineralization stages.

The chemical composition of actinolite was determined using electron probe microanalysis (EPMA; Table SM2, Supplemental Material). EPMA analysis was performed at the Electron Microbeam Analysis Laboratory, University of Michigan by using a Cameca SX-100 with a voltage of 15 keV, a current of 20 nA and a 2 µm beam. We measured Si, Ti, Al, V, Cr, Mn, Fe, Ni, Mg, Ca, Na, F and Cl. All results, counting times, standards and detection limits are listed in the Supplementary Materials.

High-resolution quantitative X-ray wavelength dispersive spectrometry (WDS) maps of representative grains from both mineralization events were acquired on a JEOL 8530F field-emission electron probe microanalyzer equipped with five wavelength-dispersive spectrometers at the Center for Microscopy, Characterization and Analysis (CMCA), the University of Western Australia, Perth, WA. The elements Si, Ti, Al, Mn, Fe, Mg, Ca, Na and Cl were measured. Detection limit maps were acquired for these elements and applied as the minimum cut-off values. Map acquisition utilized a 15 keV accelerating voltage, 100 nA beam current and a fully focused beam. Pixel dimensions were chosen between as 0.5, 1 or 2 µm^2^ depending on the size of the map area, and 150 ms per pixel dwell time. Data were processed using the Calcimage^®^ (v. 12.8.0) software package and output to Surfer^®^ (v. 8).

From the chemical compositional data the Fe# was calculated in order to trace systematical variations, and potential correlation with temperature, as the Fe# tends to decrease with increasing temperature^26,27^. Each EPMA analysis was linked to a corresponding actinolite texture at the micro-scale, (green circles in Fig. 2; Table SM2 and SM3, Supplemental Material), and the Fe# was calculated for each data point (Fig. 2; Table SM2, Supplemental Material). Textures such as core, rim, early replacement or late replacement were used to establish a temporal relationship of the variation of the Fe# in actinolite grains. The Fe# average values shown as orange in Fig. 2 were determined from the earlier paragenetic textures present in the samples (core or replacement early). In samples with no early textures, average Fe# was calculated from the remaining textures (cumulate or homogeneous).

In addition to Fe# spot calculation, we computationally converted the Fe and Mg concentrations from WDS maps of representative actinolite grains Fe# maps. Fe# maps were obtained by extracting each pixel from the WDS map as a XYZ point using Surfer (v.8) and exporting this as a .txt map. The .txt maps were then processed in Python by using the Matplotlib package^32^. For this an empty 3D stack was first created and then filled with the .txt maps for each element in the WDS maps. The 3D stack was then reshaped into a 2D array where each row contains the pixels of one element. The 2D array allowed us to work with pixels as elemental concentration data. First, we isolated the actinolite grains from the rest of the map by using Ca combined with Si concentrations. Once we had all the pixels corresponding to the actinolite grains, we calculated the Fe# using the Fe and Mg concentrations. Then we reshaped the resulting 2D data array back into a 3D one and plotted the Fe# WDS map using the matplotlib function “imshow()”. The final images obtained are those shown in Fig. 2A–E.

## Results

**Figure 3:**
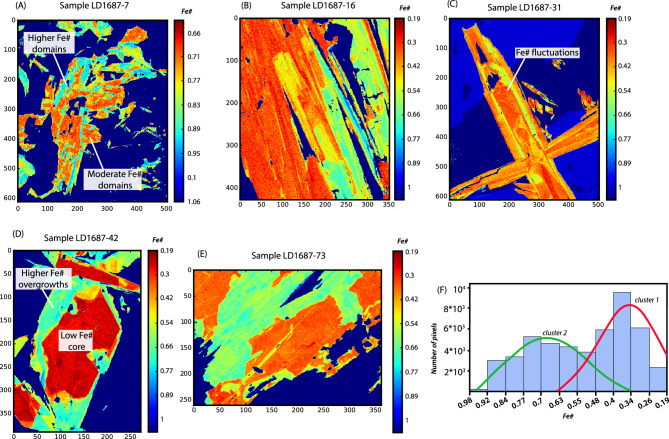
(**A**–**E)** Grain-scale Fe# maps of representative actinolite samples from drill core LD1687. (**F**) Histogram showing number of pixels vs Fe#. The images (**A**-**E**) were generated by Python (https://www.anaconda.com/download) by using the Matplotlib package^32^.

Calculated actinolite Fe# range from ~0.19 to ~1 (Figs. 2 and 3). The average Fe# calculated for earlier actinolite textures decreases with depth down the drill hole (orange stars in Fig. 2). The Fe# of later textures show a broadly positive relationship with Cu and Au grades (Fig. 2).

The maps of Fe# in actinolite (Fig. 3) reveal a complex crystallization history, with intra-crystalline variations of Fe# < 0.2 that reflect strong Fe and Mg zonations (Fig. 3A–E). In the shallow levels of the deposit (row A in Fig. 2), intra-crystalline chemical zonation of actinolite reveals domains of Fe# between 0.68 and 0.75 surrounded by replacement textures with higher Fe# (0.8 to 0.9) (Fig. 3A). Overgrowths on actinolite cores with non-equilibrium growth textures are recognized at intermediate to deeper levels (Fig. 2, rows B to E), indicating at least two phases of actinolite formation (Fig. 3B–E). In the deepest levels of the system (Fig. 2, rows D–E), the composition of actinolite cores indicate early lower Fe# (0.2 to 0.4) and overgrowths with higher Fe# (0.6 to 0.75) (Fig. 3D,E). An actinolite grain at intermediate depth alternates compositional layers that record complex fluctuations that may indicate crystallization from several hydrothermal episodes within one or more pulses (Fig. 3C).

**Figure 4:**
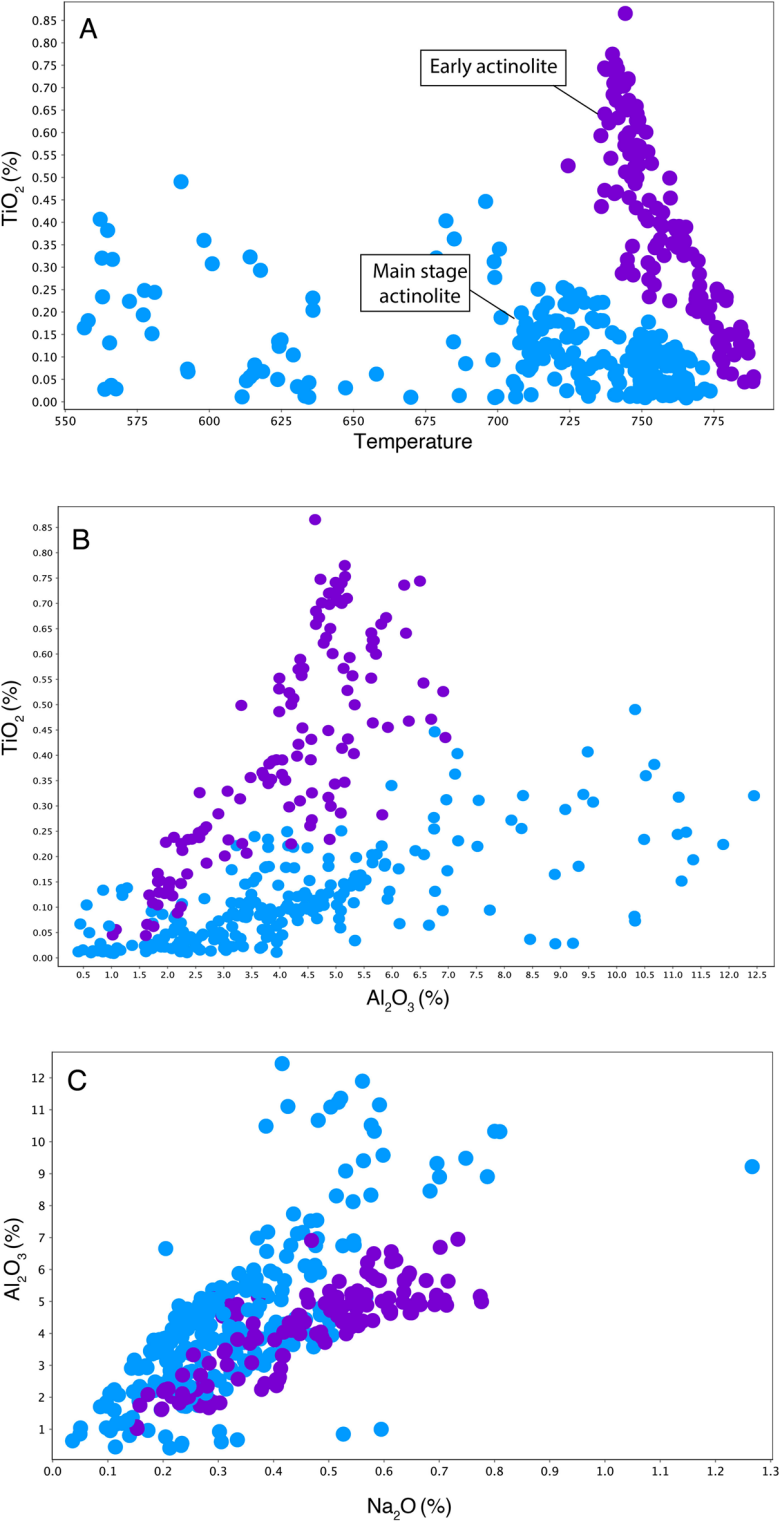
Major element variation plots with early (purple) and main-stage (blue) actinolite clusters based on Fe#, TiO_2_, Al_2_O_3_ and Na_2_O concentrations.

**Figure 5:**
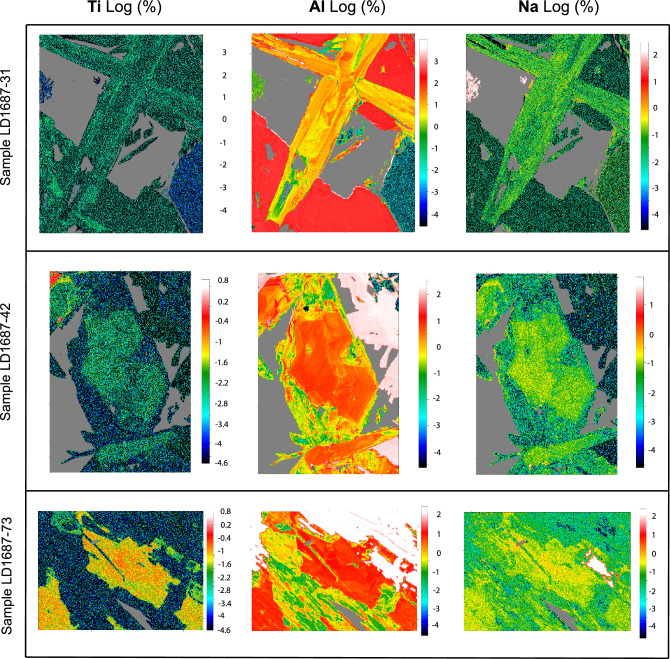
WDS compositional maps showing Ti, Al and Na variation in actinolite grains. Maps are in logarithmic scale. Titanium, Al and Na are displayed left to right and samples from top to bottom. Images were created using Surfer (v.8; https://www.goldensoftware.com/products/surfer).

The intra- and inter-crystalline grain-scale Fe# variations are represented in histograms constructed from each pixel in the Fe# maps (Fig. 3F). Two distinct main clusters are observed in Fig. 3F; a lower Fe# cluster-1 (0.63 to 0.19) and a higher Fe# cluster-2 (0.34 to 0.1). Most actinolite cores display low Fe# values (e.g. Fig. 3D; Table SM3, Supplemental Material) coincident with cluster-1. In contrast, most overgrowths are characterized by higher Fe# values coincident with cluster-2 values, and representing a later pulse (Table SM3, Supplemental Material). Besides showing distinct differences in their Fe# values, the two clusters also display differences in their major element compositions (Fig. 4) including two distinct clusters of Ti concentration (Fig. 4). Higher Ti concentrations in actinolite mostly occurs in the cores of actinolite grains at depth in early, Cu-poor samples with Fe-rich magnetite-actinolite alteration. Lower Ti concentrations are typical of actinolite from the main-stage alteration associated with Cu mineralization (Table SM4, Supplemental Material). EMPA-WDS compositional maps reveal zoning of Ti, Al and Na concentrations (in a logarithmic scale; Fig. 5).

## Discussion

### Temperature controls on mineral precipitation

The two distinct actinolite compositional clusters (Figs. 3F, 4) suggest that the Candelaria IOCG deposit formed during two distinct mineralization pulses as potentially part of one evolving hydrothermal system. These pulses correspond to a widespread lower Fe# and higher Ti content magnetite-actinolite (cluster-1) and a more localized higher Fe# and lower Ti content Cu event(s) (cluster-2). Previous research has associated lower Fe# to higher temperature formation in amphiboles^26,27^ suggesting that cluster-1 formed at a higher temperature than cluster-2. The composition of actinolite, can be sensitive to prograde and retrograde metamorphic reactions^33^, the amphibole morphology^34^, large changes in pressure conditions^35^, and the presence of coexisting minerals that can incorporate or buffer Fe and Mg^33^. In the Candelaria deposit, the composition of the host rocks is relatively uniform^6^, actinolite composition is independent of grain morphology or texture (Fig. SM2, Supplemental Material), pressure conditions are relatively constant over the sampled range of depth (~1 kbar)^21^, and there are no significant overprinting events in the area^6,8^. Therefore, we interpret the variation in the composition of actinolite to be predominantly related to changes in temperature. Differences in temperature formation are also suggested by variations of the Ti content between both clusters. Previous research has proposed that Ti concentration in Ca rich amphiboles (with a.p.f.u. between 1.2 and 2.0) increases with temperature in igneous and metamorphic environments^36–39^. The higher Ti contents of the deep and early actinolite grains suggest a higher temperature formation compared with the later, main stage actinolite grains associated with Cu mineralization. Titanium concentration in actinolite can also be affected by the coeval crystallization of magnetite^40^, which may also contribute to the observed trend between early and main-stage actinolite (Fig. 4).

**Figure 6:**
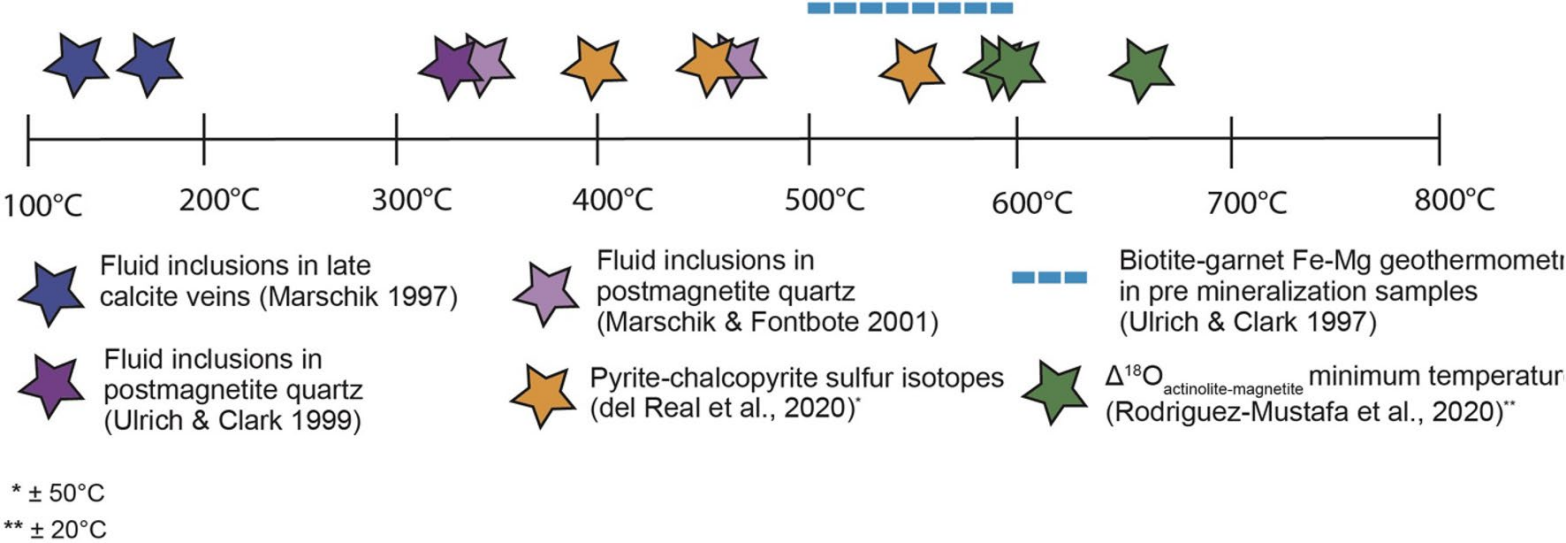
Compilation of previous temperature estimations for alteration and mineralisation in the Candelaria-Punta del Cobre IOCG district^8,20,21,28,41,42^.

At Candelaria, formation temperatures are constrained by sulphur isotopes from chalcopyrite-pyrite veins (Δ^34^S_pyrite-chalcopyrite_)^20^ associated with actinolite (e.g. Fig. 1D) and oxygen isotopes from actinolite-magnetite pairs (Δ^18^O_actinolite-magnetite_) in samples from the same drill hole^21^ as used in this study. These, and results from other workers^8,28,41,42^ suggest that Cu-Fe mineralisation and associated actinolite-rich alteration formed over a range of ~350-650 °C (Fig. 6). The early, probably higher temperature, cluster-1 pulse is related to disseminated fine grained magnetite-actinolite mineralization that is ubiquitous in the district^6,8^. The Fe content of the Candelaria system, represented mainly by the abundance of magnetite, increases systematically from top to bottom below 500 m (Fig. 2). At an estimated constant pressure of 100 MPa, Fe(II) will be transported as FeCl_2_ in a hydrothermal fluid, and will precipitate as magnetite at temperatures above ~500 °C^21,43,44^. Therefore, the formation temperatures of cluster-1 are likely > 500 °C and correlate with the early magnetite-rich pulse documented throughout the Candelaria district^6^.

In contrast, Cu grades increase at intermediate and shallow levels at Candelaria (Fig. 2) broadly correlating with cluster-2. These findings suggest that actinolite from cluster-2, often found as overgrowths on higher-temperature actinolite cores (cluster-1, e.g. Fig 2D; Table SM3, Supplemental Material), records a hydrothermal event where Cu-rich fluids cooled to temperatures below ~550 °C^45^, explaining the observed correspondence between high Cu grades and actinolites from cluster-2 (Fig. 2). The implication is that Cu precipitated as chalcopyrite as cluster-2 actinolite precipitated, once the hydrothermal fluid cooled below ~550 °C, as indicated by available sulfur isotope and limited fluid inclusion data^8,20^ and corresponding with chalcopyrite precipitation temperatures calculated experimentally^45,46^.

The two clusters of actinolite compositions are interpreted as distinct hydrothermal episodes and are consistent with data from other IOCG and IOA deposits. The chemical composition of actinolite from cluster-1 (Fig. 3F) together with previous oxygen isotopes from actinolite-magnetite pairs (Δ^18^O_actinolite-magnetite_) in samples from the same drill hole^21^ are similar to the chemistry and isotopic signatures of actinolite from Andean IOA deposits (e.g. El Romeral and Los Colorados deposits in Chile)^47,48^. The early, higher-temperature event in the Candelaria-Punta del Cobre district has a similar mineralization/alteration paragenesis to Andean IOA deposits (e.g. Cerro Negro Norte, El Romeral, Los Colorados, Marcona)^30,49–51^. Therefore, the early mineralization event in Candelaria is analogous to high-temperature Fe-rich mineralization in IOA deposits.

### Mineralization episodes triggered by tapping reservoirs of magmatic-hydrothermal fluids

**Figure 7:**
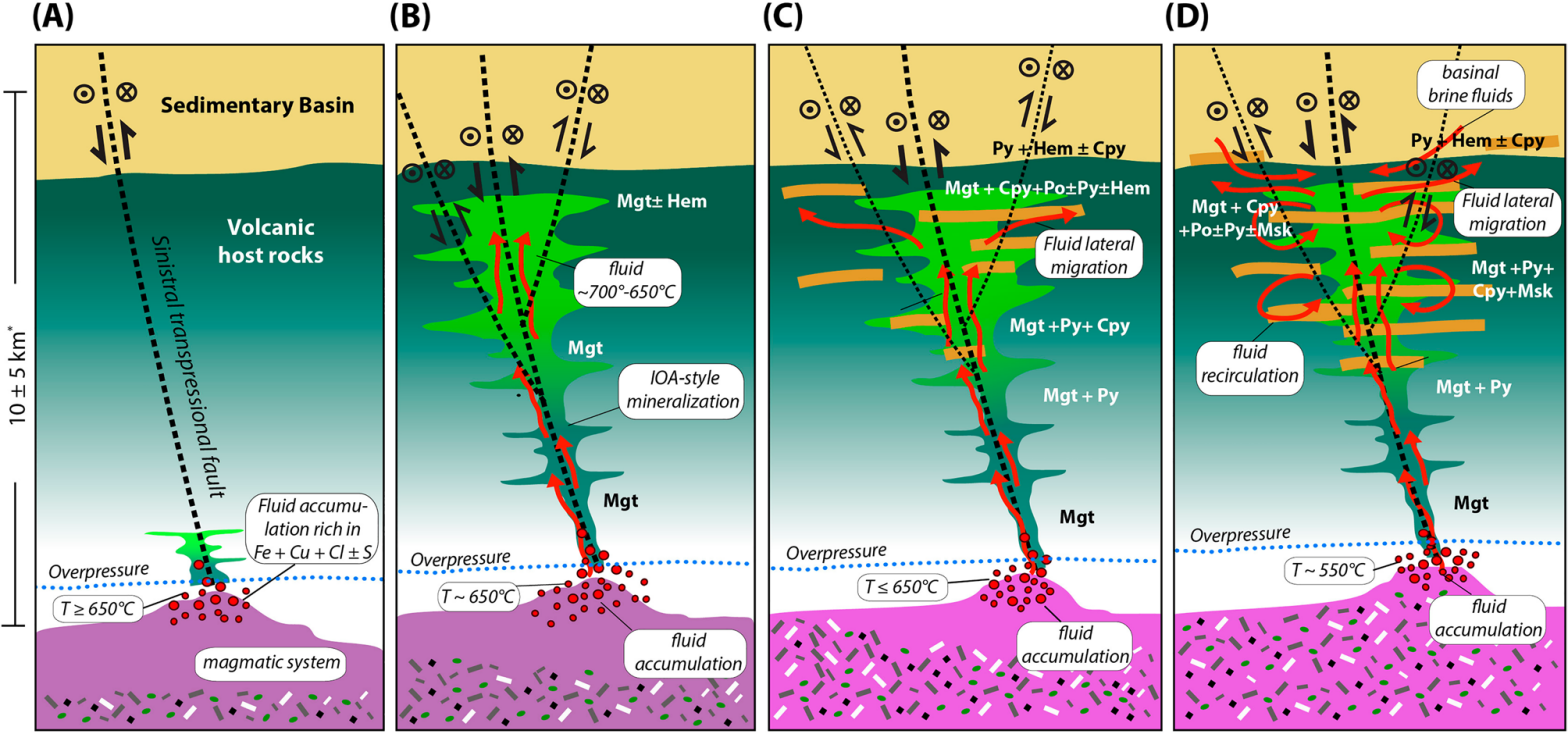
Schematic model for the Candelaria IOCG district. (**A**) Slow cooling of a dioritic intrusion and separation of a fluid phase, coalescence of the fluid phase, and encapsulation of magnetite microlites to form a magnetite-fluid suspension accumulation under overpressure conditions that scavenges Fe, Cu, Au, S and Cl^56,57^. (**B**) The accumulated fluid is sporadically “tapped” by active deep crustal sinistral strike-slip faults initially associated with the formation of the sedimentary basin. Fault movement allowed the ascent of high-temperature Fe-rich fluid. (**C**) Further fault movement would allow the ascent a (Cu, Fe)-rich fluid, Cu increases due to increase solubility of CuCl. (**D**) Further fluid cooling of the ascending hydrothermal fluids by convection causes Cu precipitation. At the final stages of the hydrothermal system hydrothermal fluids would have interacted with external, basin derived fluids which would have added reduces sulfur into the system in the form of pyrite but no significant Cu mineralization^20^. *Depth estimated from Pollard^67^ for similar hydrothermal conditions.

The observations and modeling reported here support an early, high-temperature hydrothermal pulse followed by a lower-temperature one, with magnetite and actinolite ubiquitous to both, but chalcopyrite only occurring in the latter. Figure 7 illustrates a genetic model proposed for the formation of the Candelaria deposit, which illustrates the early Fe-rich and the later Cu-rich mineralization pulse and may be applicable to the Candelaria district and other IOCG deposits. The model is consistent with geological observations and empirical data collected from other Cordilleran IOCG systems^6,8,30,52–54^. The proposed model connects the results presented here with recent models of Fe transport for IOA deposits, which are the Cu-deficient representatives of the IOCG clan^55–59^. These two styles of mineralization (IOCG and IOA) commonly overlap in time and space, and several studies have proposed a genetic link between them, with Fe-dominated IOA systems corresponding to the deeper hotter roots of Cu-rich (IOCG) systems^60–66^, although in the present model we propose that the deposits can be superimposed.

Trace element data for magnetite and pyrite together with δ^37^Cl data from the Candelaria system indicate that hydrothermal fluids in the district were sourced from intermediate to mafic silicate magmas^20,21,68^, gabbro to diorite in composition, formed in the Upper Jurassic to Lower Cretaceous magmatic arc of northern Chile^69,70^. Dioritic magmas formed under these conditions would be enriched in volatiles together with Fe, Cu, S and Cl^71,72^. Exsolution of a magmatic-hydrothermal volatile phase from the silicate melt would start during cooling of the parental magma. Volatile exsolution may be triggered by prior magnetite crystallization, as demonstrated experimentally^58,73,74^, where exsolving magmatic-hydrothermal fluid bubbles nucleate on magnetite microlites to form a magnetite-fluid suspension that rises through the intrusive body^56,74^ scavenging metals, and forming an accumulation of a metal-rich fluid in the upper part of the magma body^56^ (Fig. 7A). This zone of Fe-, Cu- and S-rich fluid accumulation, analogous to volatile concentration reported for pre-eruptive magma chambers beneath arc volcanoes and processes invoked in the formation of porphyry Cu deposits^75^, would go through periodic sealing and rupture potentially related to movement of deep crustal, sinistral strike-slip faults (Fig. 7B). Mineralization in the Candelaria district is hosted in rocks that are ca. 15 Ma older than the main Cu event^6^; therefore, the formation of structurally-related “paths” for hydrothermal fluids to ascend, i.e. the deep crustal sinistral strike-slip faults, would have been crucial for fluid flow into older volcano-sedimentary stratigraphy. In the Candelaria-Punta del Cobre district, extensional faults, initiated during the formation of the sedimentary basin that overlays the ore bodies, were inverted at the same time as hydrothermal activity^6^. Increased fault slip would rupture the carapace above crystallizing intrusions and enhance structural permeability in overlying rocks. Fluid flow would be focused by buoyancy-driven propagation through fluid-filled fractures^76^, promoting efficient ascent of the deep fluid accumulated in fractures in the upper crystallizing parts of the viscous magma, and allowing fluid redistribution in the shallow levels of the system^77–79^ (Fig. 7C). Actively deforming structures can produce pipe-like pathways linking deep reservoirs to shallow crustal levels^80,81^. The process could lead to a repetitive cycle with hydrothermal fault sealing, fluid re-accumulation, increased pressure and consequent fault rupture^82,83^. This “tapping” of the fluid reservoir would permit high temperature magmatic-hydrothermal fluids to ascend under adiabatic conditions, precipitating early magnetite^43^ (Fig. 7B) with paragenetically-related, higher-temperature actinolite (cluster-1), and generating the IOA style of mineralization similar to what has been documented for deposits such as Dominga or Marcona^29,30^.

Subsequent evolution of a distinct pulse of Cu- and S-bearing magmatic-hydrothermal fluid from the source magmatic system (Fig. 7C) and cooling of this fluid as it ascends through pre-existing superjacent structures results in precipitation of lower-temperature actinolite (cluster-2) and magnetite (Fe is still soluble at these temperatures). If the hydrothermal fluid is oxidized^20,21^, it would precipitate hematite instead of magnetite, consistent with observations in some IOCG deposits, such as Mantoverde^12,52^ (Chile) and Olympic Dam^84,85^ (Australia). Repeated, temporally distinct, fault reactivation may result in different episodes of hydrothermal activity as evidenced by actinolite micro-textures (Fig. 3A–E). Further, if the fluid reservoir is periodically recharged from fluids from the underlying magmatic system, compositionally banded textures observed in actinolite grains would record such fluid composition fluctuations (Fig. 3C).

The main Cu mineralization episode at Candelaria would have occurred when ascending fluid(s) cooled to temperatures below ~550 °C precipitating chalcopyrite, due to the sharp drop in chalcopyrite solubility^45^ (Fig. 7D). Furthermore, the presence of early magnetite might have facilitated the precipitation of Cu sulfides^86^, as suggested by geological observations and petrographic evidence of chalcopyrite replacing early magnetite in the Candelaria deposit (Fig. SM3). As cooling progresses, chalcopyrite precipitation will continue, reaching a peak at temperatures of 550–400 °C^8,20,45^. Ascending fluids may have also migrated laterally forming satellite orebodies as a result of mixing with oxidized (basinal?) fluids from the overlying basin^87^, which may have contributed additional sulfur aiding sulfide precipitation^20,21^ (Fig. 7D).

The observation of two temporally distinct hydrothermal pulses is consistent with episodic replenishment of an evolving crustal magmatic system where the magmatic-hydrothermal fluid evolves from a newly emplaced magma that either underplates previously emplaced magma or forcibly intrudes and mixes with previously emplaced magma^77,78^. Such buoyancy-driven outgassing of magmatic-hydrothermal fluid from magma can efficiently transfer fluid-soluble elements such as those found in IOCG deposits^77^. At Candelaria, the data indicate that the first fluid pulse resulted in mineralization dominated by magnetite with actinolite and minor sulfide, whereas the second fluid pulse resulted in mineralization dominated by magnetite, actinolite and Cu-Fe sulfides, where the latter make the deposit economic. These observations are consistent with the first fluid transporting Fe, Ca, Mg and Si, and the second fluid transporting the former elements and, critically, also Cu and S. The chemistry of magmatic-hydrothermal fluid exsolved from silicate melt is controlled by the pressure, temperature, oxidation state and composition of the melt from which the fluid exsolves^88,89^. The low concentration of Cu and S in the first fluid pulse, evidenced by the modally minor amount of sulfide coeval with early magnetite and actinolite, is consistent with the evolution of a magmatic-hydrothermal fluid from a magma that had lost Cu and S to a sulfide crystal/liquid which was not subsequently resorbed prior to degassing and was not resorbed during the degassing event^88,90^. The fluid responsible for the subsequent Cu-sulfide rich pulse could have outgassed from the same evolving magma body and resulted in resorption of earlier formed Cu sulfide crystal/liquid, or could have evolved from a newly emplaced magma where the released volatiles overcame capillary resistance and ascended along permeable channels developed in overlying, older crystal mush^78^. The efficient ascent of a magmatic-hydrothermal fluid in such evolving magmatic systems is transient and dependent entirely on the rate of supply of ascending magmatic-hydrothermal fluid^79^. Such intermittent degassing events plausibly explain the superimposed magnetite-actinolite and younger magnetite-actinolite-Cu-sulfide mineralization events at Candelaria. The constraint on the timing of the evolving hydrothermal system is yet to be properly evaluated, although previous research in other IOCG/IOA districts such as the Marcona-Mina Justa have estimated that millions of years could separate de Fe-rich from the Cu-rich stage^30^. Understanding the time difference between these two stages in the Candelaria-Punta del Cobre district will be essential for better characterizing the magmatic evolution of the hydrothermal fluid source responsible for mineralization.

### A new paradigm that connects IOA and IOCG mineralization

Our genetic model involving at least two distinct phases of hydrothermal activity introduces an important addition to how we understand the evolution and connection between IOA and IOCG mineralization styles. As noted, previous studies have suggested a spatial and temporal relationship between IOA and IOCG mineralization, where the IOA could represent the roots of an IOCG system^56,60,62,63,90^. Further, the presence of minor sulfides in IOA deposits (e.g. Los Colorados, El Romeral, Cerro Negro Norte)^51^ and early, pre-Cu mineralization, magnetite-actinolite in IOCG deposits (e.g. El Espino, Mantoverde, Mina Justa, Raúl Condestable)^30,52–54,91^ point to a transition between IOA and shallow IOCG systems. However, those authors assumed that both IOA and IOCG mineralization were the result of a single cooling fluid. Although this may be the case for IOA deposits and small, vein-type IOCG systems^57,61,63,64^, our data point to at least two distinct pulses of hydrothermal activity involved in the formation the Candelaria IOCG system, which are superimposed. These pulses most likely correspond to hydrothermal fluids of contrasting temperatures, and possibly with distinct metal budgets derived from an evolving magmatic source(s).

## Conclusions

The deposit-scale actinolite data presented here are used as a proxy to determine mineralization temperatures and provide a new tool to track the evolution of IOCG mineral systems. Furthermore, our results offer an explanation for how the Cu-deficient IOA-type mineralization relates spatially to Cu-rich IOCG deposits. We propose that both mineral systems can represent temporally distinct nonequivalent pulses albeit part of the same metallogenic system. We argue that IOA- and IOCG-type systems should not necessarily be viewed as related to formation depth, but rather be the result of temperature gradients and an evolving magmatic source affecting hydrothermal fluid composition and circulation. Most importantly, we argue that world-class IOCG systems are formed by distinct, episodic pulses of hydrothermal activity. The detailed understanding of the characteristics and key controls of the magmatic-hydrothermal evolution responsible for the formation of IOCG deposits, and therefore the factors that control metal transfer, is a critical step in the development of new strategies for successful exploration. Therefore, the results presented here open new opportunities for Cu exploration in districts that are historically rich in Fe.

## Supplementary Information

Supplementary Information 1.

Supplementary Information 2.

## Data Availability

The authors declare that the data supporting this study are available within the paper and its supplementary information.
