# Supplementary material for: Formation of giant iron oxide-copper-gold deposits by superimposed episodic hydrothermal pulses
Source: Sci Rep. 2023 Jul 25;13:12041. doi: 10.1038/s41598-023-37713-w (PMC10368639; doi:10.1038/s41598-023-37713-w)
Supplement: Supplementary file 1 — Supplementary Information 1. [file 41598_2023_37713_MOESM1_ESM.pdf]

Figures Supplemental Material

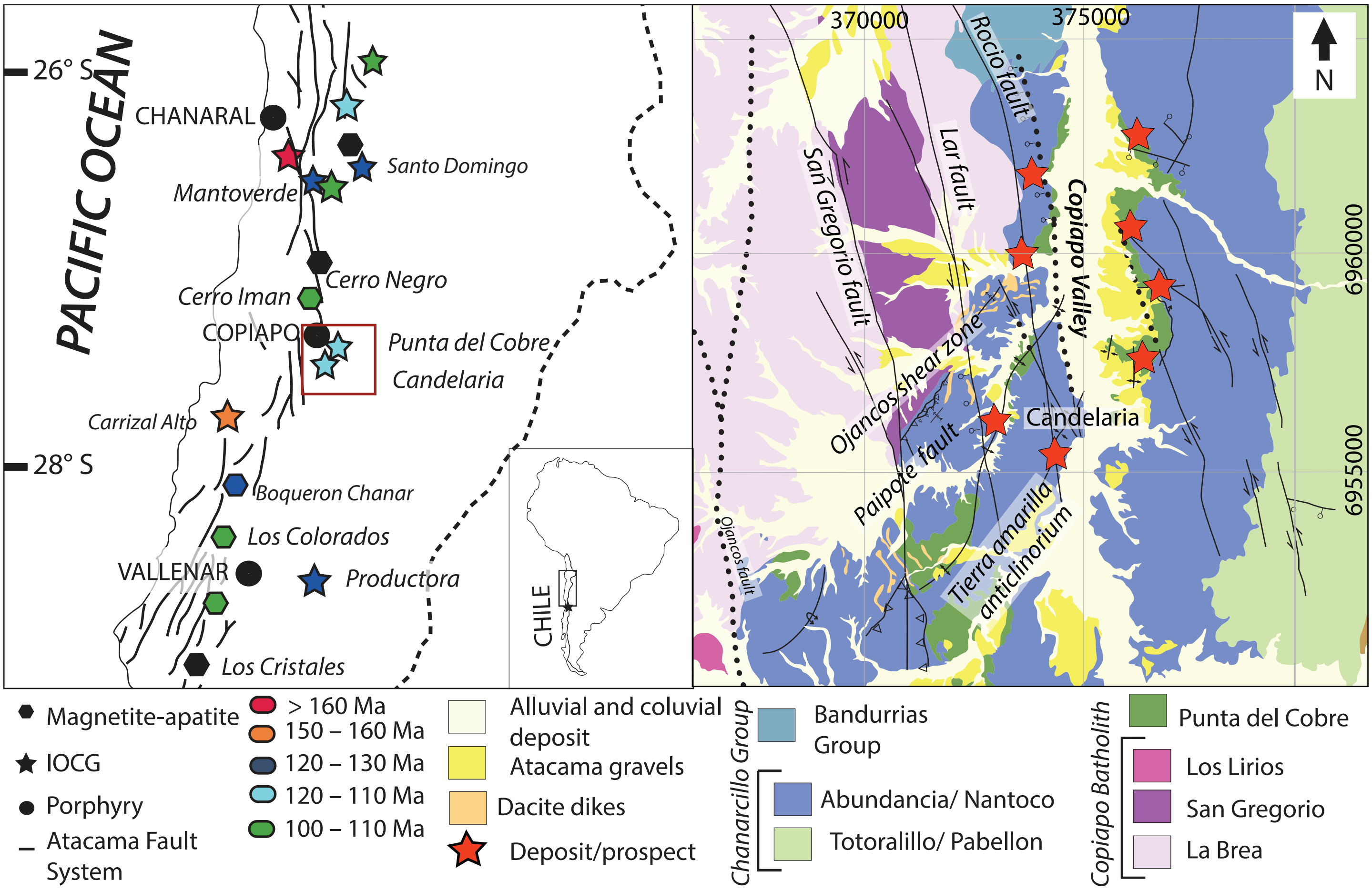

Figure SM1: Left, location of IOCG, IOA and porphyry deposits formed during the Upper Jurassic–Lower Cretaceous in the Atacama region (modified del Real et al.[1]). Right, simplified geological map of the Candelaria-Punta del Cobre district with the main IOCG deposits (modified del Real et al. [1]). Map was created by using Adobe Illustrator (v. 26.0.2)

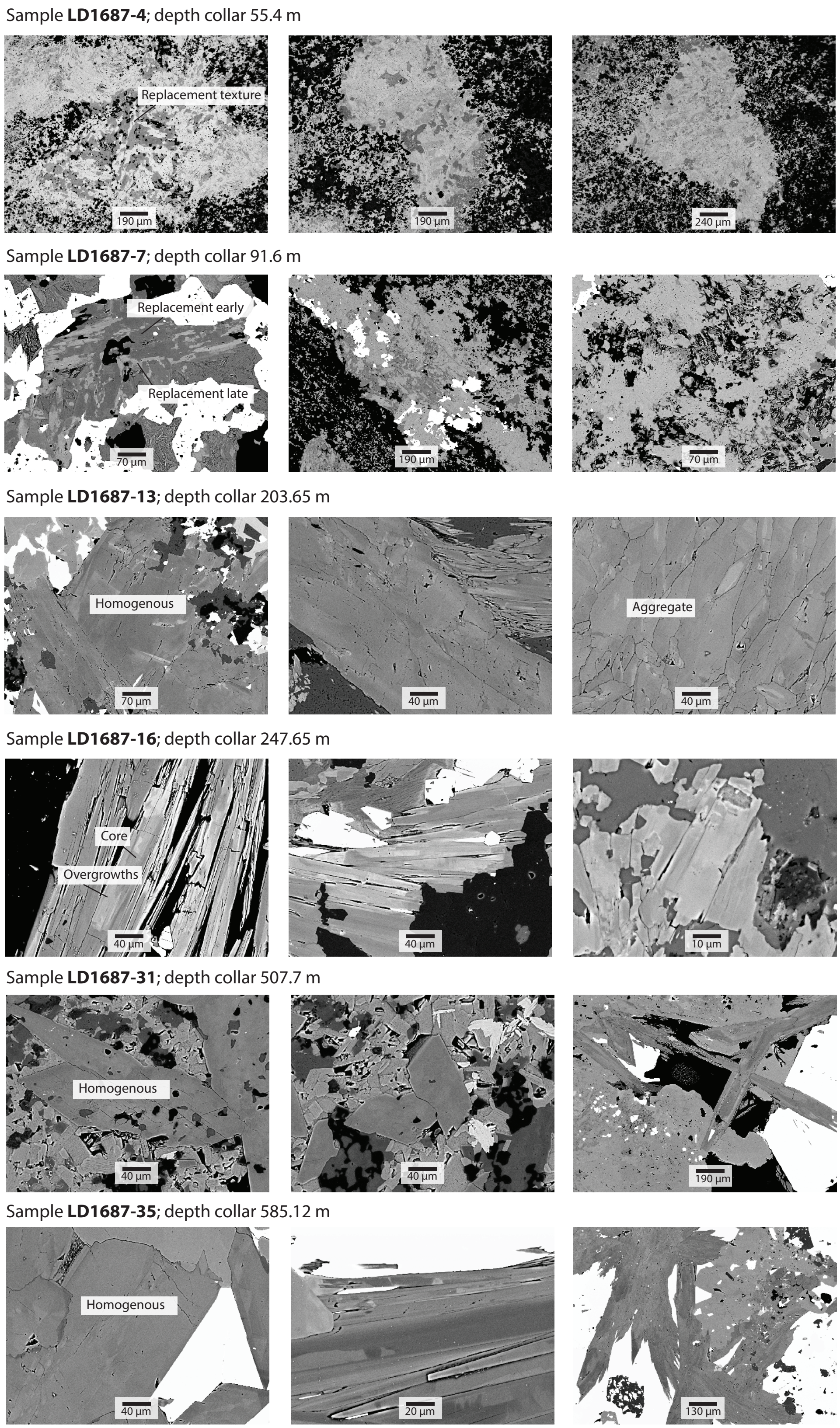

Figure SM2: BSE images of actinolite grains analyzed by EPMA. The images show the different micro-textural types, including replacements (samples LD1687-4,-7 and-73), actinolite grains with core-to-rim chemical zoning and overgrowths (samples LD1687-16, -31, -42 and -53), chemically homogenous actinolite (samples LD1687-13, -31, -35, -63, -65 and -73), and aggregates of small actinolite crystals (samples LD1687-13, -35 and -70).

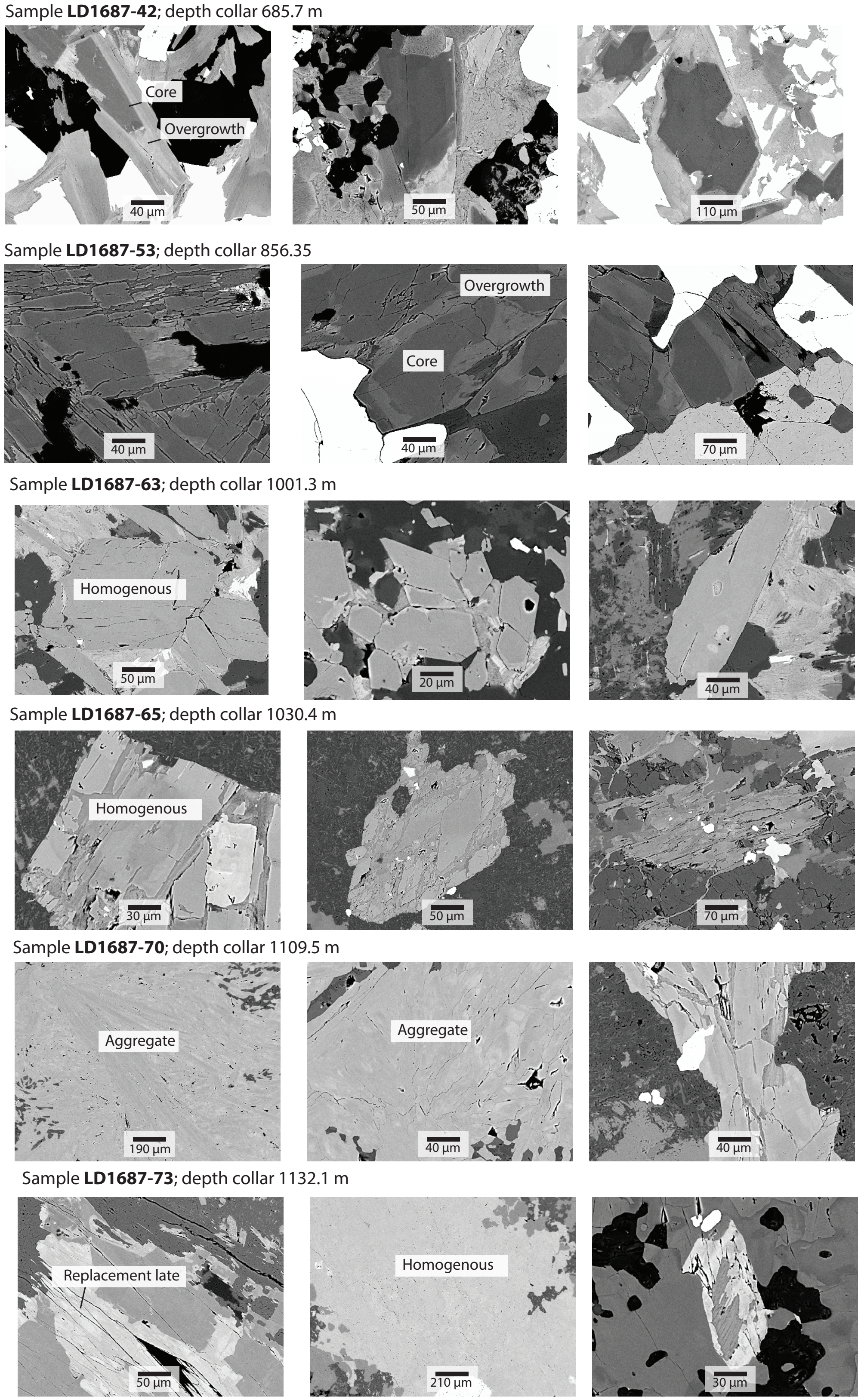

Continuation of figure SM2: BSE images of actinolite grains analyzed by EPMA. The images show the different micro-textural types, including replacements (samples LD1687-4,-7 and-73), actinolite grains with core-to-rim chemical zoning and overgrowths (samples LD1687-16, -31, -42 and -53), chemically homogenous actinolite (samples LD1687-13, -31, -35, -63, -65 and -73), and aggregates of small actinolite crystals (samples LD1687-13, -35 and -70).

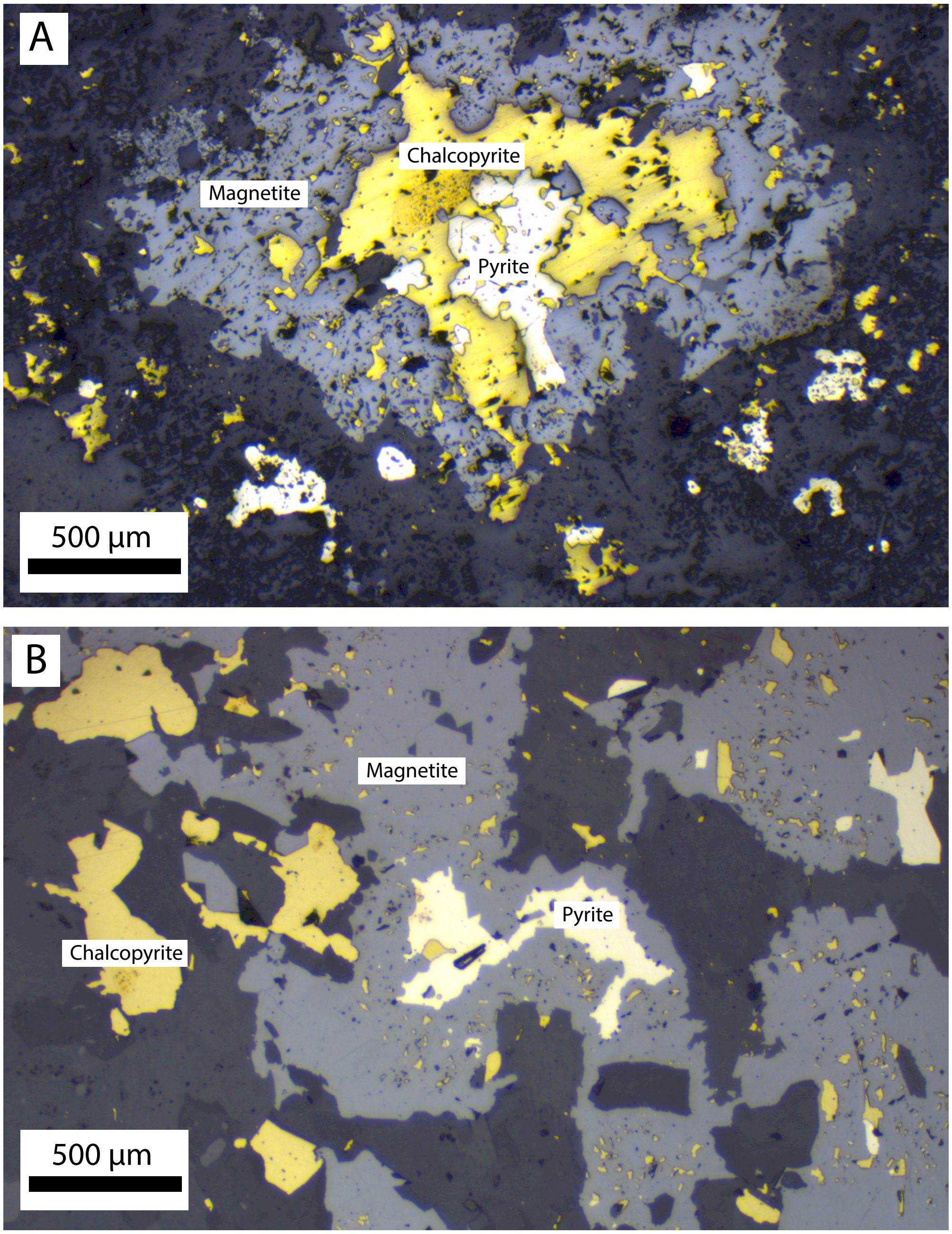

Figure SM3: Microphotographs of chalcopyrite and pyrite replacing magnetite from mineralized samples from the Candelaria district. Photo A corresponds to drill hole DH996 in the Santos deposit just east from the Candelaria deposit at a depth of 118.5 m from surface (Fig, SM1). The sample has chalcopyrite-pyrite disseminated and patchy mineralization with intense magnetite-biotite alteration and is hosted in the volcanic sedimentary unit of the Punta del Cobre Formation. Photo B corresponds to drill hole LE0011 south of the Candelaria open pit at a depth of 866.87 m from surface (Fig. SM1), The sample has chalcopyrite-pyrite disseminated with magnetite-feldspar-actinolite-biotite alteration and is hosted in the lower andesite unit of the Punta del Cobre Formation

Tables Supplemental Material

Sample	Depth from surface (m)	Host Rock	Sample Description	Actinolite description
LD1687-4	55.4	Volcanic sedimentary Unit	Volcanic tuff highly altered with pink garnet alteration with a actinolite-chlorite-diopside-biotite-pyrite vein	euhedral actinolite with replacement textures
LD1687-7	91.6	Volcanic sedimentary Unit	Volcanic sediments replaced to intercalations of magnetite, actinolite, biotite with chalcopyrite, pyrrhotite and minor pyrite	Actinolite semi-prismatic euhedral grains
LD1687-13	203.65	Lower Andesite	Lower andesite with quartz veinlets. Patches of pyrite with actinolite and surrounded by chalcopyrite-feldspar and quartz	Euhedral prismatic aggregates of actinolite
LD1687-16	247.65	Lower Andesite	Brecciated lower andesite with intense pink feldspar alteration with epidote patches. Veins or patches of actinolite-magnetite and chalcopyrite-pyrite. Epidote patches.	Euhedral bladed and prismatic cumulates of actinolite
LD1687-31	507.7	Lower Andesite	Lower Andesite with intense biotite alteration and magnetite and minor actinolite disseminated and in veins	Actinolite euhedral grains with elongated or prismatic habit
LD1687-35	585.12	Lower Andesite	Breccia with andesite clasts and chalcopyrite-pyrite-magnetite cement crosscut by pyrite-chalcopyrite-actinolite-magnetite veinlets	Euhedral bladed and prismatic aggregates of actinolite
LD1687-42	685.7	Lower Andesite	Lower Andesite with intense magnetite alteration pervasively replacing the aphanitic matrix of the host rock. Magnetite-chalcopyrite-pyrite and actinolite-sulfide-magnetite- (albite halo) veins.	Euhedral prismatic actinoite grains
LD1687-53	856.35	Lower Andesite	Porphyritic andesite with large plagioclase crystals crosscut by a pyrite- □feldspar-chalcopyrite□-actinolite vein	Euhedral prismatic actinoite grains
LD1687-63	1001.3	Lower Andesite	Andesitic breccia with pyrite-chlorite-actinolite patches and disseminated pyrite-magnetite.	Euhedral prismatic actinoite grains
LD1687-65	1030.4	Lower Andesite	Volcanic rock with patches of albite-chlorite-actinolite and titanite alteration and disseminated magnetite-pyrite-epidote-feldspar-actinolite.	Euhedral prismatic actinoite grains
LD1687-70	1109.5	Lower Andesite	Andesite with disseminated magnetite and actinolite aggregates.	Aggregate of euhedral actinolite
LD1687-73	1132.1	Lower Andesite	Andesite with disseminated magnetite and minor actinolite. Actinolite-magnetite veins with minor sulfides.	Aggregate of euhedral actinolite

Table SM1: Sample location and description. Drill hole LD1687, located in near the center of the Candelaria mine pit. UTM coordinates are east 73430 and north 55991; elevation 474 m a.s.l.; an azimuth of 244° and a dip of -60°.

Table SM2: EPMA results, detection limits, actinolite textures and Fe#

Actinolite texture	N	Fe#	Si [wt.%]	Al [wt.%]	Fe [wt.%]	Mn [wt.%]	Ti [wt.%]	Mg [wt.%]	Ca [wt.%]	Na [wt.%]	K [wt.%]	Cl [wt.%]
Core	76	0.31	***24.57***	***1.71***	***9.54***	***0.18***	***0.15***	***9.59***	***8.92***	***0.21***	***0.11***	***0.04***
Rim	68	0.42	***23.92***	***1.36***	***12.58***	***0.29***	***0.08***	***7.95***	***8.78***	***0.21***	***0.09***	***0.04***
Replacement Early	21	0.68	***23.4***	***1.06***	***20.22***	***1.14***	***0.18***	***4.00***	***8.28***	***0.17***	***0.19***	***0.22***
Replacement Late	19	0.85	***17.66***	***5.87***	***22.20***	***1.28***	***0.11***	***1.52***	***8.12***	***0.38***	***2.09***	***2.52***
Cummulate	67	0.38	***24.55***	***1.59***	***11.42***	***0.17***	***0.17***	***8.41***	***8.82***	***0.26***	***0.12***	***0.10***
Homogeneous	194	0.34	***23.89***	***2.19***	***10.45***	***0.23***	***0.16***	***9.01***	***8.84***	***0.24***	***0.18***	***0.06***

N= number of analyses

Table SM3: Median results for major elements analyzed by EPMA and Fe# calculations for each texture described for the actinolite grains used in this study

Actinolite Type		Fe#	Si [wt.%]	Ti [wt.%]	Al [wt.%]	Fe [wt.%]	Mn [wt.%]	Mg [wt.%]	Ca [wt.%]	Na [wt.%]	K [wt.%]	Cl [wt.%]	F [wt.%]
Lower Fe# (0.48-0.19)	min-max	0.48-0.22	21.4-26.4	bdl-0.59	0.1-4.01	7.24-14.24	0.066-1.065	6.78-11.09	7.08-9.35	0.012-0.57	0.007-0.36	bdl-0.75	bdl-0.27
	median	***0.33***	***24.2***	***0.11***	***1.98***	***10.38***	***0.22***	***9.1***	***8.3***	***0.22***	***0.14***	***0.05***	***0.07***
	N/bdl	365/0	365/0	322/43	365/0	365/0	365/0	365/0	365/0	365/0	365/0	23/342	105/260
Higher Fe# (0.91-0.48)	min-max	0.91-0.48	16.42-22.35	0.006-0.38	0.174-7.29	14.23-24.57	0.21-0.172	1.03-6.79	7.7-15.9	0.026-0.93	0.01-2.88	bdl-3.54	bdl-0.147
	median	***0.48***	***22.4***	***0.09***	***2.02***	***20.26***	***0.49***	***3.9***	***8.4***	***0.28***	***0.24***	***0.22***	***0.05***
	N/bdl	86/0	86/0	86/0	86/0	86/0	86/0	86/0	86/0	86/0	86/0	79/7	53/30

N=number of analyses, bdl= number of analyses below detection limit

Table SM4: Range of results and mendian values for major elements obtaines by EPMA analysis of the actinolite grains used for this study separated by Fe# cluster.

References Supplemental Material

1. Sillitoe, R. H. Iron oxide-copper-gold deposits: An Andean view. *Miner. Depos*. **38**, 787–812 (2003).

2. Arévalo, C. The Coastal Cordillera/Precordillera Boundary in the Tierra Amarilla area (27 20’-27 40’S/70 05’-70 20’W), northern Chile, and the structural setting of the. Unpubl. PhD, Kingst. Univ., Kingston-Canada (1999).

3. Tiling, R. I. El Batolito andino cerca de Copiapó, Provincia de Atacama. Geología y Petrología. *Andean Geol*. **3**, 1–24 (1976).
